# The Validity of Physiological Measures to Identify Differences in Intrinsic Cognitive Load

**DOI:** 10.3389/fpsyg.2021.702538

**Published:** 2021-09-10

**Authors:** Paul Ayres, Joy Yeonjoo Lee, Fred Paas, Jeroen J. G. van Merriënboer

**Affiliations:** ^1^School of Education, University of New South Wales, Sydney, NSW, Australia; ^2^School of Health Professions Education, Maastricht University, Maastricht, Netherlands; ^3^Department of Psychology, Education and Child Studies, Erasmus University, Rotterdam, Netherlands; ^4^School of Education/Early Start, University of Wollongong, Wollongong, NSW, Australia

**Keywords:** intrinsic cognitive load, physiological measures, validity, working-memory load, workload, cognitive load theory

## Abstract

A sample of 33 experiments was extracted from the Web-of-Science database over a 5-year period (2016–2020) that used physiological measures to measure intrinsic cognitive load. Only studies that required participants to solve tasks of varying complexities using a within-subjects design were included. The sample identified a number of different physiological measures obtained by recording signals from four main body categories (heart and lungs, eyes, skin, and brain), as well as subjective measures. The overall validity of the measures was assessed by examining construct validity and sensitivity. It was found that the vast majority of physiological measures had some level of validity, but varied considerably in sensitivity to detect subtle changes in intrinsic cognitive load. Validity was also influenced by the type of task. Eye-measures were found to be the most sensitive followed by the heart and lungs, skin, and brain. However, subjective measures had the highest levels of validity. It is concluded that a combination of physiological and subjective measures is most effective in detecting changes in intrinsic cognitive load.

## Introduction

The main aim of this study was to examine the validity of using physiological techniques to measure cognitive load by examining construct validity (see Gravetter and Forzano, 2018) and sensitivity (see Longo and Orru, 2018). More specifically to investigate the ability of physiological measures to detect differences in intrinsic cognitive load caused by tasks of varying complexity. To meet this aim we examined the findings from a number of studies drawn from a 5-year sample that measured cognitive load using physiological techniques. In particular, we were interested in examining a sample of contemporary studies that had access to the most up-to-date technology.

Researchers across many fields have been interested in the amount of mental resources invested in attempting a task. One such field is human factors, where studies have focused on everyday tasks such as driving a motor vehicle. In particular, of much interest has been the mental resources required to not only drive the car but deal with other requirements or distractors. Measurement of these mental resources has received much attention, for example, Wickens (2002) developed a model based on multiple resource theory that predicted dual-task interference.

A number of descriptors have been used to represent these types of mental investments such as working memory load, mental workload, and cognitive load, according to the context used and/or the theoretical influences of the researchers. Although such labels are often used interchangeably and have similar meanings, we will use the term cognitive load because of its close association with cognitive load theory (CLT) and the identification of different types of cognitive load (see Sweller et al., 2011), which is an important consideration in the current paper.

Cognitive load theory emerged in the 1980s as a theory that made predictions about learning and problem solving based on the amount of mental resources and effort (cognitive load) invested in the tasks. An integral part of CLT (see Sweller et al., 2011; Sweller et al., 2019) has therefore been to find instruments that measure cognitive load in order to provide direct evidence for the assumptions made and to advance the theory further. Up until now a number of self-rating subjective measures (see Paas, 1992; van Gog and Paas, 2008; Ayres, 2018) have been developed as the preferred methods. However, despite their widescale use, many researchers have argued for more objective alternatives such as working memory dual-tasks (see Park and Brünken, 2015) or physiological measures (see Antonenko and Niederhauser, 2010).

In more recent times, helped by advances and the availability of new technologies, physiological measures have become more popular in CLT research (see Ayres, 2020). Many physiological measures have already been used based on theoretical arguments and experimental data to confirm individual validity (see Kane, 2013). However, our study was a broader investigation to gain a more global overview of physiological measures in order to gain an up-to-date picture of their validity.

Cognitive load theory has identified three types of cognitive load: intrinsic, extraneous, and germane (see Sweller et al., 2019). According to CLT intrinsic cognitive load is generated by the element interactivity (elements that need to be processed simultaneously) of the task (Sweller et al., 2011); whereas in instructional settings, cognitive load can also be generated by learners dealing with the instructional designs (extraneous cognitive load) and the actual learning processes generated (germane cognitive load). Complex tasks typically have many interacting elements creating high levels of intrinsic cognitive load. The intrinsic cognitive load generated by any task is not fixed *per se* as it is dependent upon the expertise of the problem-solver or learner. Learners with high levels of expertise possess knowledge structures (schemas) that enable them to chunk together many elements (see Chi et al., 1982; Sweller et al., 2011) thus reducing intrinsic cognitive load. It should be noted that CLT emphasizes the importance of interacting elements for creating complexity in learning settings; however, increased cognitive load does not always depend on interactivity for other non-learning types of tasks. For example, trying to recall 12 random numbers after a brief observation requires more mental effort than recalling five numbers. Nevertheless, interactivity between elements can be a major cause of intrinsic cognitive load.

Subjective measures have been reasonably successful by requiring learners to rate different aspects of the learning process using multi-item scales (see Leppink et al., 2013), although it is debatable whether learners are able to identify different forms of cognitive load when many cognitive processes are interacting. In contrast, as far as we know no physiological measures have been developed to distinguish the types of cognitive load due to a number of confounding factors such as interactions between task complexity and learning or stress. To make a consistent analysis by controlling for possible confounding factors, the present study focused on studies that generated changes in intrinsic cognitive load caused by variations in problem complexity rather than by additional learning factors. By focusing on studies that require participants to solve tasks without instruction, and no requirement for learning, cognitive load can be narrowed down to one source (task complexity). Under these conditions we assume total cognitive load to be equivalent to intrinsic cognitive load as no instruction or learning, or other interacting factors are directly involved (see Ayres, 2006). Hence for our sample of studies, we expected the various physiological techniques used to measure only intrinsic load as no other types of cognitive load were present.

Mental arithmetic tasks provide a good example of tasks that vary in complexity and generate different levels of intrinsic cognitive load. They require the simultaneous storage and processing of information, which generates working memory load (cognitive load). Because completing a task serves as a different function to learning about the task, the source of cognitive load is intrinsic and dependent upon element interactivity only, as no instructional steps are included. Consider the following two problems: (a) calculate 3^∗^4 + 7; and (b) 12^∗^8 + 14 + 11^∗^2. The first problem is fairly straightforward with only two simple calculations involved; whereas the latter has four more difficult calculations with greater element interactivity. The second problem requires more working memory resources to overcome the intrinsic cognitive load generated.

From a validity perspective, Borsboom et al. (2004) argue that “A test is valid for measuring an attribute if: (a) the attribute exists; and (b) variations in the attribute causally produce variation in the measurement outcomes” (p. 1061). Clearly, cognitive load exists for mental arithmetic tasks (and all tasks that require working memory load), so any instrument that purports to measure cognitive load should find differences in cognitive load between the two problems described above. Based on cognitive load theory we hypothesize that within-participants comparisons of tasks with different levels of complexity reveal differences in intrinsic load that should become visible in physiological measures. The more studies support this hypothesis for a specific physiological measure, the higher the construct validity of this measure (Gravetter and Forzano, 2018).

Borsboom et al. (2004) also suggest that detecting changes in the attribute is integral to validity. Using Messick’s argument that validity (see Messick, 1989) does not simply have black or white outcomes, Borsboom et al. (2004) suggest that there can be different levels of validity dependent upon how sensitive the measure is to detecting changes. Longo and Orru (2018) refer to an ability to detect changes as *sensitivity*. Hence, we consider sensitivity as a second important feature in establishing the validity of a physiological measure. Note that the concept of validity can encompass the concept of sensitivity, since validity in general is a broader concept. Thus, we treat both construct validity and sensitivity as aspects of validity.

As previously mentioned many studies have used physiological techniques to measure cognitive load. Four main categories have been identified that correspond with major body organs: the heart (i.e., cardiovascular) and lungs (i.e., respiratory), eyes, skin (i.e., electrodermal), and brain. The following sections summarize some of the key ways that data has been extracted from these four categories to measure cognitive load.

## Cardiovascular and Respiratory Measures of Cognitive Load

### Heart Rate

Cardiovascular measures or heart rate (HR) parameters have a long history as psychophysiological indices of cognitive load (Paas et al., 2003). However, HR data can be problematic because it is affected by many psychological and physiological factors, such as emotions and physical activities (Jorna, 1992). The challenge has been to disentangle these factors to isolate the factors that can be indicative of cognitive load or use well-controlled experimental designs. An example of a well-controlled design can be found in the study by Mehler et al. (2012), who showed that HR is a highly sensitive physiological measure for detecting systematic variations in cognitive load.

### Heart Rate Variability

Blitz et al. (1970) showed that heart rate variability (HRV) can be used to differentiate between different levels of cognitive load. HRV can be assessed through measurement of the electrical activity of the heart, which can be visualized in an electrocardiogram (Mulder, 1992), or measurement of blood volume changes in the microvascular bed tissue using a light-based technology called photoplethysmography (Challoner, 1979).

According to the Task Force of the European Society of Cardiology and the North American Society of Pacing Electrophysiology (1996), HRV can be described as the oscillation in the interval between consecutive heartbeats as well as the oscillations between consecutive instantaneous HRs (i.e., variability in time between the successive R-tops of the cardiogram). In a study on the usefulness of HRV as an index of operator effort, Aasman et al. (1987; see also Mulder, 1992) further specified the HRV measure based on the knowledge that the time between successive heartbeats is determined by three different feedback mechanisms, connected with respiration, blood pressure (BP), and body temperature regulation. Using a special mathematical technique (i.e., spectral analysis) to investigate periodical components of the HRV, Aasman et al. (1987) were able to show that cognitive load is specifically related to the short-term regulation of arterial BP. The relationship between cognitive load and HRV is indirect (Solhjoo et al., 2019), because an increase in cognitive load will lead to an increase in BP, which will lead to a decrease in HRV. A similar indirect relationship has also been identified for respiratory activity with increasing cognitive load increasing the respiratory frequency (e.g., Grassmann et al., 2016b), which will lead to a decrease in HRV (e.g., Song and Lehrer, 2003). The spectral analysis technique can be used to separate the effects of respiratory rate (high frequency band, 0.15–0.40 Hz) and thermoregulation (low-frequency band, 0.02–0.06 Hz) from the mid-frequency band (0.07–0.14 Hz), which is determined by the arterial BP regulation and related to cognitive load.

The HRV measure is generally accepted as a measure of cognitive load (e.g., Finsen et al., 2001; De Rivecourt et al., 2008; Thayer et al., 2012). However, Paas et al. (1994) have argued that it has mainly been used successfully with short-duration basic task (e.g., binary decision tasks) under well-controlled conditions. Paas et al. (1994) showed that with longer-lasting learning tasks typically used in educational research, the validity and sensitivity of the spectral analysis technique of the HRV was low. The technique was only sensitive to relatively large differences in cognitive load, i.e., differences between mentally inactive and mentally active periods. According to Aasman et al. (1987) the high intrinsic variability in the HR signal is one of the sources of its low reliability and relative insensitivity to small differences in processing load between tasks. The studies that Paas et al. (1994) analyzed to determine the sensitivity of the HRV technique used relatively long duration learning tasks. In contrast to basic tasks, which mainly consist of mentally active periods, such tasks naturally contain both mentally active and inactive periods, and consequently create a rather noisy signal. In addition, although spectral analysis of HRV allows cognitive load to be measured at a higher rate than subjective measures, it cannot be considered a real-time measure, because it needs time to process. From this real-time measurement perspective, HR can also be argued to have an advantage over HRV, because HR changes can be detected in a much shorter period than changes in HRV.

### Respiratory Measures

In contrast to cardiovascular measures, the use of respiratory measures has received much less research attention (for a review, see Grassmann et al., 2016a). Similar to the cardiovascular measures, respiratory measures are also influenced by and reflective for metabolic, psychological, and behavioral processes (Wientjes et al., 1998). Grassmann et al. (2016a) reviewed studies that used respiratory indices of cognitive load as a function of task difficulty, task duration, and concurrent performance feedback.

Research into the relationship between respiratory measures and cognitive load has used measures based on time (e.g., number of breaths per minute, i.e., respiration rate), volume (amount of air inhaled during one respiratory cycle, i.e., tidal volume), gas exchange (e.g., proportion of released CO_2_ to inhaled O_2_, i.e., respiratory exchange ratio), and variability parameters of these measures. In their review, Grassmann et al. (2016a) found that those respiratory parameters can be measured with breathing monitors, respiratory inductive plethysmography, strain gauges, impedance-based methods, capnography, and metabolic analyzers. Results of this study revealed that cognitive load was positively related to respiration rate (e.g., Backs and Seljos, 1994), and frequency of sighing (e.g., Vlemincx et al., 2011). In addition, negative relationships with cognitive load were found for both inspiratory and expiratory time (e.g., Pattyn et al., 2010) and partial pressure of end-tidal carbon dioxide (petCO_2_; Grassmann et al., 2016a). For the respiratory measures it is important to note that they can be disrupted by verbal activities, such as effects of talking on the breathing pattern, which can be a confound when using breathing pattern as an index of cognitive load (e.g., Tininenko et al., 2012).

## Eye Activity Measures of Cognitive Load

For decades, eye-tracking indices have been used to measure cognitive load in various fields (Rosch and Vogel-Walcutt, 2013; Glaholt, 2014; Wilbanks and McMullan, 2018). Thanks to its portability and unobtrusiveness, eye tracking supports more natural task environments (Eckstein et al., 2017). Moreover, its indices correspond to not only autonomic responses (e.g., pupil dilation, blinks) but also consciously modulated processes (e.g., eye fixation), facilitating the investigation of diverse indicators of cognitive load. The following sections describe some key measures of cognitive load drawn from eye data and important conditions.

### Pupil Dilation

Pupil dilation reflects noradrenergic activity of the autonomous nervous system that regulates arousal and mental activity (Eckstein et al., 2017). A large number of studies have shown that pupil dilation is positively correlated with cognitive demands imposed by the tasks (Hess and Polt, 1964; Kahneman and Beatty, 1966; Hyönä et al., 1995; Van Orden et al., 2001). Most modern image-based eye-trackers can readily collect pupil data. However, multiple factors (e.g., light reflex, gaze position, pupil dilation latency) can affect this data, which challenges proper measurement of the cognitive effects on pupil size. Thus, researchers must take extra precautions to establish well-controlled experimental setups, for instance, maintaining a constant luminance of stimuli, using baseline data, or employing computational correction methods (Hayes and Petrov, 2016; Chen et al., 2017).

### Blink Rate

The frequency of spontaneous blinks, or blink rate, is modulated by dopaminergic activity in the central nervous system that involves goal-oriented behavior and reward processing (Eckstein et al., 2017). Studies have shown that blink rate significantly increases as a function of time on task, fatigue, and workload (Stern et al., 1994; Tsai et al., 2007). However, this measure for assessing cognitive load is task-specific. When the task requires intensive visual processing (e.g., reading, air traffic control), blink rate is rather inhibited, resulting in a decreased rate (Van Orden et al., 2001; Recarte et al., 2008). Blinks can be easily detected by eye trackers, while several artifacts (e.g., reflections in glasses, participant motion) must be regulated (Holmqvist and Andersson, 2017, Chapter 15). Moreover, blink data is not continuous and its distribution is often non-Gaussian, requiring auxiliary calculation methods (Siegle et al., 2008).

### Fixation

Eye fixation is a more consciously modulated behavior compared to pupil dilation and blinks. Three types of fixation measures have been frequently used for assessing cognitive load, reflecting different aspects of visual information processing: fixation rate (the number of fixations divided by a given time), fixation duration (time span when the eye is relatively still), and transition rate (the number of gaze shifts per second from one area of interest to another). Note that the first two, fixation rate and duration, are inversely correlated given the same trial duration, which makes the interpretation of the results highly task-specific. For instance, if the task requires frequent searching of different locations (e.g., scene perception, surveillance), increased fixation rate is associated with high cognitive load, accompanying short fixation duration (Van Orden et al., 2001; Chen et al., 2018). If the task includes deep and effortful processing of particular visual targets, long fixation duration would indicate high cognitive load (Callan, 1998; Henderson, 2011; Reingold and Glaholt, 2014).

When the task involves integration of information between different areas of interest (AOIs), transition rate can be a suitable measure (Schmidt-Weigand et al., 2010). Studies have shown that high cognitive load increases transition rate in static task environments, while it decreases the rate in dynamic task environments (van Meeuwen et al., 2014; Lee et al., 2019). For fixation data analysis, data quality and AOI definition are critical. Valid fixations should be detected after assuring data quality in terms of accuracy, precision, and tracking loss (Orquin and Holmqvist, 2018). Researchers should then carefully define AOIs relevant to sources of cognitive load, and select the measures pertinent to given task characteristics through task analysis and piloting.

### Other Eye Measures

More eye-tracking and ocular indices have been explored in various research contexts. Variability in horizontal gaze position reduced as cognitive load increased in driving simulation tasks (He et al., 2019). In a simulated surgical task, intraocular pressure (i.e., fluid pressure inside the eye) was positively correlated with cognitive load (Vera et al., 2019). Ocular astigmatism aberration (i.e., deviation of optic elements of the eye), mediated by the intraocular pressure, was also shown to increase as a function of cognitive load (Jiménez et al., 2018). Since different measures can demonstrate different aspects of cognitive load, combining multiple eye measures may provide a higher construct validity and sensitivity as a cognitive load measure (Van Orden et al., 2000; Ryu and Myung, 2005; Mehler et al., 2009).

## Electrodermal Measures of Cognitive Load

Electrodermal measures have a long history as psychophysiological measures of emotional or cognitive stress and arousal (for a review see Posada-Quintero and Chon, 2020). The basis of the measurement of electrodermal activity (EDA) is the change in electrical activity in the eccrine sweat glands on the plantar and palmar sides of the hand, which are particularly responsive to psychological stimuli imposing stress. Increased stress leads to increased sweating, which lowers the resistance and augments the electrical conductance of the skin (Dawson et al., 2000).

Within the EDA signal, two components can be distinguished. Firstly, a tonic skin conductance component (i.e., skin conductance level) that changes slowly over time. This component is considered a measure of psychophysiological activation. Secondly, a phasic skin conductance component (i.e., skin conductance response) that changes abruptly. These fast changes are reflected in the peaks in the electrodermal signal and are also called the galvanic skin response (Braithwaite et al., 2013). This component is impacted by stress and arousal (e.g., Hoogerheide et al., 2019).

Based on the knowledge that cognitive load is one of the cognitive states that causes people to experience stress it is assumed that changes in cognitive load affect the galvanic skin response through changes in skin conductance, with increases in cognitive load leading to increases in the galvanic skin response. Several studies have confirmed the positive relation between cognitive load and the galvanic skin response (e.g., Setz et al., 2009; Nourbakhsh et al., 2012; Larmuseau et al., 2019). Mehler et al. (2012), who investigated the sensitivity of skin conductance level to cognitive load, studied three age groups (20–29, 40–49, 60–69) working on a working memory task at three levels of cognitive load. They found a significant pattern of incremental increase of skin conductance level as a function of increasing cognitive load, thereby confirming the sensitivity of the measure of skin conductance level.

Vanneste et al. (2020) recently argued that the usability of EDA measures (i.e., skin conductance response rate and skin conductance response duration) as measurement instrument for cognitive load is limited, because it could only explain a limited proportion of the variance in cognitive load (approx. 22%). Charles and Nixon (2019) have suggested that EDA may be sensitive to sudden, but not gradual changes in cognitive load. However, this is only seems to apply to skin conductance response measures, because skin conductance level measures have been shown to increase with a gradual changes in cognitive load (e.g., Mehler et al., 2012).

Skin conductance response activity is commonly measured by the frequency of the peaks (i.e., skin conductance response rate), the duration of the peaks (skin conductance response duration), and the magnitude of the peaks (i.e., skin conductance magnitude) in the signal. Recently, Posada-Quintero et al. (2016) have introduced the spectral analysis technique to process skin conductance response activity data. This analysis method is commonly used to investigate periodical components of the HRV signal (e.g., Aasman et al., 1987). The newly developed index, incorporating the components between 0.08 and 0.24 Hz, was found to be highly sensitive to cognitive stress.

## Brain Activity Measures of Cognitive Load

In the past, measures of activity of the brain have mainly been used to assess cognitive load in controlled laboratory settings because they required advanced, unportable equipment for electroencephalography (EEG) or functional magnetic resonance imaging (fMRI). But nowadays, the use of brain measures is becoming more popular because new apparatus, such as wireless EEG caps and portable fNIRS devices (functional near infrared spectroscopy) are mobile, easy to use, and less obtrusive.

### Electroencephalography

Electroencephalography records electrical activity of the brain. Multiple electrodes are placed on the scalp (typically using the 10/20 system; Jasper, 1958) and measure voltage fluctuations resulting from ionic current within the neurons of the brain. Assessments typically focus on the type of neural oscillations (‘brain waves’) that can be observed in EEG signals in the frequency domain. Spectral analysis gives insight into information contained in the frequency domain, distinguishing waveforms such as gamma (>35 Hz), beta (12–35 Hz), alpha (8–12 Hz), theta (4–8 Hz), and delta (0.5–4 Hz). Electrodes are placed on different locations of the scalp so that they read from different lobes or regions of the brain: Pre-frontal, frontal, temporal, parietal, occipital, and central. Cognitive load has mainly been found to be correlated with an increase in the parietal alpha band power and frontal-midline theta band power (Antonenko et al., 2010).

### Functional Magnetic Resonance Imaging

Functional magnetic resonance imaging measures brain activity by detecting changes associated with cerebral blood flow, which is directly coupled to neuronal activation. Typically, it uses the blood oxygen level dependent (BOLD) contrast, which images the changes in blood flow related to energy use of brain cells. Statistical procedures are necessary to extract the underlying signal because it is frequently corrupted by noise from various sources. The level of brain activation in the whole brain or its specific regions can be graphically represented by color-coding its strength (e.g., showing fMRI BOLD signal increases in red and decreases in blue). Cognitive load has been found to be correlated with increased activation of neural regions associated with working memory, such as the fronto-parietal attention network (e.g., Tan et al., 2016; Mäki-Marttunen et al., 2019).

### Functional Near Infrared Spectroscopy

Functional near infrared spectroscopy is an optical brain monitoring technique which uses near-infrared spectroscopy to measure brain activity by estimating cortical hemodynamic activity which occurs in response to neural activity. The signal can be compared with the BOLD signal measured by fMRI and is capable of measuring changes both in oxy- and deoxyhemoglobin concentration from regions near the cortical surface; local increases of oxyhemoglobin as well as decreases in deoxyhemoglobin are indicators of cortical activity. A typical system contains pairs of optical source and detector probes that are placed on the scalp with a lightweight headband typically using the same locations as EEG electrodes (i.e., the 10/20 system). As for fMRI, cognitive load is correlated with increased activation of the fronto-parietal network (Hosseini et al., 2017). In addition, using a combination of EEG and fNIRS signals has been shown to improve the sensitivity of cognitive load measurements (Aghajani et al., 2017).

## Research Questions

Our aim was to gain an overview of the validity of physiological measures of intrinsic cognitive load from a collection of contemporary studies by examining construct validity and sensitivity. In particular, we were interested in identifying the different types of measures in use in such studies within the four main categories identified above: namely the heart and lungs, eyes, skin, and brain. The more studies that find the expected effects for a specific physiological measure, the stronger the support for its construct validity. Also by comparing the various measures with each other we aimed to identify any variations in levels of sensitivity. Furthermore, as we assumed that such variations could be influenced by specific tasks, we also investigated how validity was influenced by the types of tasks used. Our main research questions were:

RQ1: Do the physiological measures have construct validity in detecting changes in intrinsic cognitive load across tasks?

RQ2: How sensitive are the physiological measures to detecting changes in intrinsic cognitive load?

RQ3: Does the type of task impact on overall validity?

## Materials and Methods

In the present study we included only experiments that had within-subject designs (*Criterion 1*) comparing tasks with different levels of complexity (*Criterion 2*). Criterion 1 ensures that potential moderating factors such as prior knowledge that can impact on intrinsic cognitive load were limited. Criterion 2 ensured that intrinsic cognitive load would vary across tasks and thus could be detected by a valid physiological measure. Consistent with the main focus of this study we only included experiments that actually measured cognitive load using physiological measures (*Criterion 3*). In order to have a sufficiently large sample based on contemporary studies we examined data from the last 5 years. Although there are many databases, we chose the Web-of-Science as it is one of the most prestigious and authentic, and provided a sufficient enough sample to fulfill our aims.

### Selecting the Sample of Studies

#### Step 1

To find an up-to-date sample of studies that featured physiological measures of cognitive load, a search was conducted in the *Web of Science* using the keywords “Physiological measures cognitive load” for the previous 5 years up until 30 November 2020. This search included articles, book chapters, and books. Some slight variations of the keywords were used such as working memory load, which produced little if any differences. In total 208 studies were initially identified.

#### Step 2

The abstract of each source was read to filter out any study that clearly did not meet our essential criteria of physiological measures of cognitive load (Criterion 3), within-subject designs (Criterion 1) with problems of varying complexity (Criterion 2). This analysis narrowed down the sample to 98 studies.

#### Step 3

From Step 2 it was possible to eliminate many studies that clearly did not meet Criterion 1–3, but many abstracts did not have sufficient information to make a definitive decision. Hence, a more thorough reading and analysis of each study was conducted to ensure each condition was satisfied. In particular to satisfy Criterion 2 it was necessary to include only studies that found significant differences on performance scores across the tasks. Using the example given above, recalling 12 random numbers after a brief observation is more mentally demanding than recalling five numbers. It is expected that more errors would be made recalling the 12-number task than the 5-number task, and this difference in errors would be caused by variations in cognitive load generated by complexity rather than prior knowledge about the domain. Hence, studies that did not report such significant differences between tasks were excluded.

A small number of studies were included that did not report score differences because this information was provided in previous studies or based on expert opinion (*N* = 3), or time to completion (*N* = 1). Studies that manipulated factors such as anxiety, stress, and other confounding factors were also eliminated (*N* = 4) as these factors are known to impact on working memory. For example, pupil dilation can indicate emotional arousal and therefore indicate extraneous cognitive load caused through distraction if the emotion is not part of the task (Lee et al., 2020).

These processes led to a final sample of 28 studies with 33 experiments (note: these studies are starred in the reference section). The mean participant size per experiment was 29.2 (*SD* = 14.8) with 53% males; 26 of the experiments consisted of adults with a mean age between 20 and 30, five included adults with no recorded mean statistics, and two studies focused on older adults (mean ages of 58 and 70).

### Data Analysis

For each study a record was made of: (a) what cognitive load measures were used; (b) the type of tasks used; (c) the number of within-subject tasks; and (d) how many significant differences were found on performance tasks and cognitive load measures, and if these differences matched each other. It was notable that nearly all studies included a number of different measures of cognitive load.

#### Physiological Measures of Cognitive Load

The physiological measures as expected could be grouped into four categories consistent with the major organs of the body: the heart and lungs, eyes, skin, and brain. It was notable that 62.5% of all studies used measures from one category, 28.1% from two categories, 6.3% from three categories, and 3.1% from all four categories, indicating a battery of tests were utilized. In each of the four categories different types of measures were used, often in the same study. For example, a study might record both HR and HRV. Results for each category are described next.

##### Heart and Respiration Measures

Information (see Table 1) was collected from ECGs (*N* = 5), HR monitors (*N* = 3), a fNIRS (*N* = 1), a BP monitor (*N* = 1), a plethysmograph (*N* = 1), breathing monitors (*N* = 2), and a multi-purpose Shimmer GSR + device (*N* = 1). The main heart measures used were HR and HRV. There was also vascular response index measure used by calculating the ratio of specific amplitudes of the signals. Respiration rates and BP were also used. Eleven studies included heart and respiration measures, eight of which recorded different types of measurement within this category.

**TABLE 1:** Heart and respiration measures.

**Tasks**	**Type of measure**	**No. of experiments**	**At least 1 significant difference**	**Matches with task performance**	**Pair-wise deviations**
**Arithmetic**					
	HR	1	1 (100%)	1 (100%)	0
	HRV	1	1 (100%)	1 (100%)	0
	Vasc. Response	2	2 (100%)	2 (100%)	0
	BP	1	0 (0%)	0 (0%)	1.00
	Total	5	4 (80%)	4 (80%)	0.20
**Simulations**					
	HR	4	4 (100%)	3 (75%)	–0.25
	HRV	3	0 (0%)	0 (0%)	–1.67
	Respiration	3	2 (67%)	2 (67%)	–0.33
	Total	10	6 (60%)	5 (40%)	–0.70
**Memory**					
	HR	1	1 (100%)	1 (100%)	0
	HRV	3	3 (100%)	3 (100%)	0
	Respiration	1	1 (100%)	1 (100%)	0
	Total	5	5 (100%)	5 (100%)	0
**Word tasks**					
	HR	1	0 (0%)	0 (0%)	–3.00
	HRV	1	0 (0%)	0 (0%)	–3.00
	Total	2	0 (0%)	0 (0%)	–3.00
**All**					
	HR	7	6 (86%)	5 (71%)	–0.57
	HRV	8	4 (50%)	4 (50%)	–1.00
	Vasc. response	2	2 (100%)	2 (100%)	0
	Respiration	4	3 (75%)	3 (75%)	–0.25
	BP	1	0 (0%)	0 (0%)	–1.00
	Totals	22	15 (68%)	14 (64%)	–0.64

##### Eye Measures

Information (see Table 2) was collected from eye-tracking devices (*N* = 11), EEG (*N* = 1), EOG (*N* = 1), a web-camera (*N* = 1), and from tonometry (*N* = 1). The two most frequent measures in this category were pupil diameter and blink rates. The former is based on increased size of pupil averaged over time and the latter the number of blinks per time period. There were also measures of gaze fixations, saccades (gaze transitions), astigmatism, and ocular pressure. Fifteen studies included eye measures, five of which recorded different types of measurement connected to this category.

**TABLE 2:** Eyes.

**Tasks**	**Type of measure**	**No. of experiments**	**At least 1 significant difference**	**Matches with task performance**	**Pair-wise deviations**
Arithmetic Simulations	–	–	–	–	–
	Pupil diam.	6	3 (50%)	3 (50%)	–0.80
	Blink rate	4	4 (100%)	4 (100%)	0
	Fixations	3	3 (100%)	3 (100%)	+0.33
	Ocular press.	1	1 (100%)	1 (100%)	0
	Total	14	11 (79%)	11 (79%)	–0.29
Memory	–	–	–	–	–
	Pupil diam.	1	1 (100%)	1 (100%)	0
	Astigmatism	1	1 (100%)	1 (100%)	0
	Saccades	1	1 (100%)	1 (100%)	0
	Total	3	3 (100%)	3 (100%)	0
Objects/shapes	Pupil diam.	3	3 (100%)	2 (67%)	–0.67
	Blink rate	1	1 (100%)	1 (100%)	0
	Total	4	4 (100%)	3 (75%)	–0.50
Word tasks	–	–	–	–	–
	Pupil diam.	1	1 (100%)	1 (100%)	0
	Blink rate	1	1 (100%)	0 (0%)	1
	Total	2	2 (100%)	1 (50%)	–0.50
All	–	–	–	–	–
	Pupil diam.	11	8 (73%)	7 (64%)	–0.60
	Blink rate	6	6 (100%)	5 (83%)	–0.17
	Other	6	6 (100%)	6 (100%)	0
	Totals	23	20 (87%)	18 (78%)	–0.30

##### Skin Measures

Eight studies (see Table 3) collected GSR data measuring EDA from sensors attached to the foot (*N* = 1), hand (*N* = 4), fingers (*N* = 2), or wrist (*N* = 1). This data was difficult to divide into sub-categories because studies often did not provide sufficient information or used a variety of different signal features. For example, the majority of studies did not indicate which of the EDA signal components (phasic and tonic; see Vanneste et al., 2020) were measured. Although, it could be assumed that the phasic data was most likely used because of the shorter time intervals involved. Furthermore, several studies reported mean and accumulation GSR statistics based on different features such as amplitudes, total power, gradients, peak numbers, spectral density, and wave rises. Even though they had some common labels such as GSR-mean, it was not necessarily means of the same data. Some studies also transformed their data using Fourier analysis techniques. Hence, it was not possible to form consistent subgroups and therefore all GSR data was grouped together under the heading Skin-GSR.

**TABLE 3:** Skin measures.

**Tasks**	**Type of measure**	**No. of experiments**	**At least 1 significant difference**	**Matches with task performance**	**Pair-wise deviations**
Arithmetic	GSR	6	6 (100%)	3 (50%)	–1
Simulation	GSR	1	1 (100%)	1 (100%)	+1
Memory	GSR	2	1 (50%)	1 (50%)	–0.50
Objects/shapes	GSR	2	1 (50%)	1 (50%)	–0.50
Written	GSR	2	2 (100%)	2 (100%)	0
	Totals	13	11 (85%)	8 (62%)	–0.54

In two studies skin temperature was recorded. Although we note that changes in temperature due to stress or arousal can be caused by vasoconstrictions (see Vanneste et al., 2020) and therefore could be considered under the Heart and Respiration category, we reported this measure here in the Skin category because of the direct reference to, and measurement of, this part of the body. We did not include this measure in the skin category summary because it did fit easily with the other measures, which all were based on GSR signals. Its exclusion provides a more meaningful grouping for further analysis.

##### Brain Measures

*Thirteen* studies (see Table 4) reported measures based on the five sub-bands (alpha, beta, gamma, delta, and theta) of brain electrical activity obtained from EEGs (*N* = 10), fMRIs (*N* = 2), and an fNIRS (*N* = 1) data and was recorded according to the five sub-bands (see Table 4). Typically, band power or amplitude was recorded following power spectrum density analysis. Eleven studies recorded more than one sub-band of data. In some studies information was collected on various lobes of the brain such as the frontal, occipital, and parietal lobes. In these cases, data was classified according to the sub-bands (e.g., alpha). In some limited studies, signal data were combined or transformed (e.g., alpha and theta data were combined) and this was recorded under the category ‘Other.’

**TABLE 4:** Brain measures.

**Tasks**	**Type of measure**	**No. of experiments**	**At least 1 significant difference**	**Matches with task performance**	**Pair-wise deviations**
Arithmetic	Alpha	1	1 (100%)	1 (100%)	0
	Beta	1	1 (100%)	0 (0%)	–2.00
	Theta	1	1 (100%)	0 (0%)	–1.0
	Total	3	3 (100%)	1 (33%)	1.00
Simulations	Alpha	5	4 (80%)	2 (40%)	–0.80
	Beta	5	3 (60%)	0 (0%)	–1.20
	Gamma	3	2 (67%)	1 (33%)	–1.33
	Delta	3	2 (67%)	1 (33%)	–1.33
	Theta	4	2 (50%)	1 (25%)	–1.25
	Other	2	0 (0%)	0 (0%)	–2.00
	Total	22	13 (59%)	5 (23%)	–1.36
Memory	Alpha	2	2 (100%)	2 (100%)	0
	Gamma	1	1 (100%)	1 (100%)	0
	Theta	1	0 (0%)	0 (0%)	–3.00
	Other	1	1 (100%)	1(100%)	0
	Total	5	4 (80%)	4 (80%)	–0.60
Objects/shapes	Alpha	1	0 (0%)	0 (0%)	–3.00
	Beta	1	1 (100%)	0 (0%)	–2.00
	Other	2	2 (100%)	1 (50%)	–1.00
	Total	4	3 (75%)	1 (25%)	–1.50
All	Alpha	9	7 (78%)	5 (56%)	–0.78
	Beta	7	5 (71%)	0 (0%)	–1.43
	Gamma	4	3 (75%)	2 (50%)	–1.00
	Delta	3	2 (67%)	1 (33%)	–1.33
	Theta	6	3 (50%)	1 (17%)	–1.50
	Other	5	3 (60%)	2 (40%)	–1.00
	Totals	34	23 (68%)	11 (32%)	–1.15

#### Subjective Measures of Cognitive Load

Even though our main aim was to investigate physiological measures of cognitive load, many studies in the sample also included subjective measures. In particular, the NASA-TLX scale (see Hart and Staveland, 1988) was often used as a comparative tool as it was considered the gold standard in measuring workload in human–computer interaction studies. This scale requires task participants to subjectively rate: mental demand, physical demand, temporal demand, performance, effort, and frustration. All studies (*N* = 12) that used this instrument aggregated the six items to get an overall mental workload rating, which we documented. Many studies also recorded and analyzed the six sub-scales separately. In these cases, we also recorded the data for mental demand and effort, as they more closely resembled the single-item rating scales used in cognitive load theory. Further, some studies (*N* = 8) also used single-item measures of effort, difficulty, and demand, that were not part of the NASA-TLX survey, and more consistent with the scale devised by Paas (1992). This data was also recorded and included in Table 5 under the heading Single item. Although one exception to this was an aggregated measure of intrinsic cognitive load using 3 items based on the survey developed by Leppink et al. (2013).

**TABLE 5:** Subjective measures.

**Tasks**	**Type of measure**	**No. of experiments**	**At least 1 significant difference**	**Matches with task performance**	**Pair-wise deviations**
Arithmetic	Single item	3	3 (100%)	3 (100%)	0
Simulations	NASA-overall	8	7 (88%)	5 (63%)	–0.38
	NASA-effort	6	5 (83%)	3 (50%)	–0.50
	NASA- demand	5	5 (100%)	4 (100%)	0
	Total	19	17 (89%)	12 (63%)	–0.32
Memory	NASA-overall	2	2 (100%)	2 (100%)	0
	NASA-effort	1	1 (100%)	1 (100%)	0
	NASA- demand	2	2 (100%)	2 (100%)	0
	Single item	1	1 (100%)	1 (100%)	0
	Total	6	6 (100%)	6 (100%)	0
Objects/shapes	Single item	4	4 (100%)	4 (100%)	0
All	NASA-overall	10	9 (90%)	7 (70%)	–0.30
	NASA-effort	7	6 (86%)	4 (57%)	–0.43
	NASA- demand	7	7 (100%)	6 (86%)	0
	Single item	8	8 (100%)	8 (100%)	0
	Totals	32	30 (94%)	25 (78%)	–0.19

#### Types of Tasks

For each type of cognitive load measure, a record was made of the type of tasks used in the study. These could be categorized into arithmetic, working memory, simulations, object-shape manipulations and word tasks. Individual analysis of task types was recorded in Tables 1–5. Arithmetic tasks were constructed from mental arithmetic problems; simulations used specialized equipment to mimic (simulate) real-life tasks that involved motorcar driving, engineering skills, military exercises and surgery; memory tasks included n-back and digit-span tasks; object/shapes included visual object tracking and shape construction tasks; and word tasks were based on both reading and writing tasks.

### Assessing Validity

The following steps were conducted to assess the validity of the identified measures. The first step was to confirm whether the various physiological measures were capable of detecting *any* significant differences in cognitive load across the different tasks in each study. Based on cognitive load theory, it is predicted that higher complexity tasks yield higher intrinsic load that should thus be reflected in the physiological measure; the more studies provide evidence for that prediction, the higher the construct validity of the measure. Therefore, for each use of a physiological measure, a record was made of whether a significant difference was found across the tasks. This information was recorded in Column-4 (labeled *At least 1 significant difference*) for Tables 1–5. For example, Column-4 in Table 1 for HR measures collected during simulation tasks, indicates that significant differences between tasks in HR were found in four of the four experiments (100%).

The next step was to document to what extent the cognitive load measures matched the performance test scores, providing information on levels of construct validity and sensitivity. In studies that feature tasks of different complexities, it is assumed that performance scores will vary in accordance with cognitive load. As more demand is made on working memory, less correct answers would be expected (see Ayres and Sweller, 1990). For example, if a study used 3 tasks (T1, T2, and T3) there are three possible pair-wise comparisons (T1–T2, T1–T3, T2–T3). If the three tasks had significantly different levels of complexity then it would be expected that test scores would give three significant differences in pair-wise comparisons. It would then be expected that the measures of cognitive load would also detect three significant differences, consistent with the nature of the physiological measure (e.g., as task complexity increases and scores decrease, HR increases). In the case of three significant test differences and three corresponding cognitive load differences in an experiment, a match was recorded. If the cognitive load measure only found two significant differences this was considered a non-match. This information was recorded in Column-5 (labeled *Matches with task performance*) for Tables 1–5. For the simulation-HR example in Table 1 (Column-5), cognitive load measures matched test scores in three of the four studies (75% matches), indicating that for the other study cognitive load measures failed to find as many differences as the test scores.

It should be noted that in two studies in the sample no pair-wise comparisons were made, only an overall ANOVA was conducted. In these cases, only one overall comparison could be made. On some rare occasions, the cognitive load measure found more significant differences than the test performance scores suggesting a more sensitive measure, so these were also considered a match because they found at least the same number of differences.

To measure the variations between the test scores and the cognitive load scores, the differences between them were recorded. For example, if the test scores indicated three significant pairwise comparisons, but the cognitive load measure only found one, the variation was recorded as –2. For each sub-category of measure, this information was averaged and recorded in Column-6 (labeled *Pair-wise deviations*) for each table. For example, –1.67 is recorded for HRV for simulations in Table 1. Over the three studies (each individual experiment counted separately if studies contained multiple experiments) total variations short of what was expected summed to five giving an average of –1.67. On the rare occasions that the cognitive load measure found more significant differences than the test scores, it was possible to have a positive (+) average (see Table 2: fixations for simulations).

The data reported in columns 4–6 enabled differences between the sensitivity of the various measures to be compared. For example, if for three different cognitive tasks measure-A records no cognitive load differences, then scores in columns 4–6 would be recorded as (0, 0, –3). Whereas if a more sensitive measure-B records two cognitive load differences then it would be recorded as (1, 0, –1). B is clearly more sensitive than A which is reflected in this scoring rubric. Note a perfect match of three significant differences in cognitive load would be recorded as (1, 1, 0).

Finally, to make some comparisons between the different measures, overall summaries are reported in Table 6.

**TABLE 6:** Summaries.

**Tasks**	**Type of measure**	**No. of collections**	**At least 1 significant difference**	**Matches with task performance**	**Pair-wise deviations**
Arithmetic	Subjective	3	3 (100%)	3 (100%)	0
	Skin-GSR	6	6 (100%)	3 (50%)	–1.00
	Brain	3	3 (100%)	1 (33%)	–1.00
	Heart	5	4 (80%)	4 (80%)	0.20
	Eyes	–	–	–	–
	All	17	16 (94%)	11 (65%)	–0.59
Simulations	Skin-GSR	1	1 (100%)	1 (100%)	+1.00
	Subjective	19	17 (89%)	12 (63%)	–0.32
	Eyes	14	11 (79%)	11 (79%)	–0.29
	Heart	10	6 (60%)	5 (40%)	–0.70
	Brain	22	13 (59%)	5 (23%)	–1.36
	All	66	48 (73%)	34 (52%)	–0.70
Memory	Subjective	6	6 (100%)	6 (100%)	0
	Heart	5	5 (100%)	5 (100%)	0
	Eyes	3	3 (100%)	3 (100%)	0
	Brain	5	4 (80%)	4 (80%)	–0.60
	Skin-GSR	2	1 (50%)	1 (50%)	–0.50
	All	20	18 (90%)	18 (90%)	–0.20
Objects/shapes	Subjective	4	4 (100%)	4 (100%)	0
	Eyes	4	3 (75%)	3 (75%)	–0.50
	Brain	4	3 (75%)	1 (25%)	–1.50
	Skin-GSR	4	1 (25%)	1 (25%)	–1.25
	Heart	–	–	–	–
	All	16	11 (69%)	9 (56%)	–0.81
Word	Skin-GSR	2	2 (100%)	2 (100%)	0
	Eyes	2	2 (100%)	1 (50%)	–0.50
	Heart	2	0 (0%)	0 (0%)	–3.00
	Subjective	–	–	–	–
	Brain	–	–	–	–
	All	6	4 (67%)	3 (50%)	–1.17
All	Subjective	32	30 (94%)	25 (78%)	–0.19
	Eyes	23	20 (87%)	18 (78%)	–0.30
	Skin-GSR	13	11 (85%)	8 (62%)	–0.54
	Heart	22	15 (68%)	14 (64%)	–0.64
	Brain	34	23 (68%)	11 (32%)	–1.05

## Results

### Analysis of Individual Measures

#### Heart and Respiration Measures

Heart and respiration measures were recorded in 11 experiments (see Rendon-Velez et al., 2016; Wong and Epps, 2016; Reinerman-Jones et al., 2017; Wu et al., 2017; Lyu et al., 2018; Alrefaie et al., 2019; He et al., 2019; Jaiswal et al., 2019; Ahmad et al., 2020; Digiesi et al., 2020; Gupta et al., 2020; Zakeri et al., 2020). For this category (see Table 1), the most common forms of measures were HR and HRV. Respiration rates and blood pressure (BP) were also measured along with some novel indices, such as the Vascular response index, calculated from the ratio of different amplitudes taken from a photoplethysmogram waveform (see Lyu et al., 2018). For 8 of the 11 studies, more than one sub-category of measures were collected. With such a small sample size it was not possible to conduct meaningful statistical tests between individual measures; however, some observations could be made. Firstly, HR, the vascular response index and respiration rates produced high levels of consistency with test results (indicating more sensitivity), even though the two latter cases involved a small number of cases. Secondly HR measures were more sensitive than HRV measures, the latter being totally ineffective on simulation tasks. From the perspective of task type, memory and arithmetic tasks produced high levels of consistency, whereas the more complex simulations did not. The two studies with word tasks produced no significant cognitive load measures for HR or HRV. Combined across the different tasks, the heart measures were capable of finding at least one significant difference in cognitive load in 68% of studies, with exact matches of 64%.

#### Eye Measures

Eye measures were recorded in 15 experiments (see Mazur et al., 2016; Rendon-Velez et al., 2016; Wong and Epps, 2016; Hosseini et al., 2017; Yan et al., 2017; Jiménez et al., 2018; Alrefaie et al., 2019; He et al., 2019; Hossain et al., 2019; Vera et al., 2019; Ahmad et al., 2020; Maki-Marttunen et al., 2020; van Acker et al., 2020; Vanneste et al., 2020; Zakeri et al., 2020). The two most popular measures were pupil diameter and blink rates. Measuring the number of saccades, astigmatism, gaze positions, and ocular pressure were also used in a small number of experiments. For five of the 15 studies, more than one sub-category of eye measures were collected. Overall, blink rates had a very high level of being able to detect changes in cognitive load indicating greater sensitivity; whereas pupil diameter was less successful, especially on simulation tasks compared with other measures. Combined across the different tasks, the eye measures were capable of finding at least one significant difference in cognitive load in 87% of studies, with exact matches of 78%.

#### Skin Measures

Skin measures were reported in 10 experiments (see Nourbakhsh et al., 2017; Ghaderyan et al., 2018; Lyu et al., 2018; He et al., 2019; Hossain et al., 2019; Larmuseau et al., 2019; Gupta et al., 2020; Vanneste et al., 2020). Six of the 10 studies used multiple skin measures. The two most frequent forms of GSR-accumulation measure and GSR-other were able to detect differences in cognitive load at the 100% level. In contrast, GSR-average found cognitive load differences in only 50% of the time. In terms of exact matches, GSR-other had a success rate of 83% suggesting a high level of sensitivity, compared with 50% (GSR-average) and 33% (GSR-accumulation). The two cases where skin temperature was recorded found no cognitive load differences. Overall, the more infrequent measures of signal data (e.g., wave rises) produced the best results.

#### Brain Measures

Brain measures were reported in 13 experiments (see Mazur et al., 2016; Tan et al., 2016; Wang et al., 2016; Hosseini et al., 2017; Reinerman-Jones et al., 2017; Wu et al., 2017; Katahira et al., 2018; He et al., 2019; Abd Rahman et al., 2020; Gupta et al., 2020; Maki-Marttunen et al., 2020; Vanneste et al., 2020). Eleven of the 13 studies recorded multiple types of signals (e.g., alpha, beta, and gamma) that were often taken from different parts of the brain. As can be seen from Table 4, the overall brain signal measures were only able to detect differences in cognitive load at the 68% level and could only match performance differences at 32%. Measures of alpha and gamma waves were the most promising. Beta waves were able to detect differences at the 71% level but had zero matches with test scores. Delta and theta waves generally had low levels of sensitivity.

#### Subjective Measures

Subjective measures were recorded in 18 studies (see Rendon-Velez et al., 2016; Nourbakhsh et al., 2017; Reinerman-Jones et al., 2017; Yan et al., 2017; Wu et al., 2017; Ghaderyan et al., 2018; Jiménez et al., 2018; He et al., 2019; Jaiswal et al., 2019; Larmuseau et al., 2019; Vera et al., 2019; Abd Rahman et al., 2020; Digiesi et al., 2020; Gupta et al., 2020; van Acker et al., 2020; Zakeri et al., 2020). As can be seen from Table 5, subjective measures were very successful in identifying differences in cognitive load. In terms of identifying any changes in cognitive load, accuracy scores ranged from 86–100%. In terms of exact matches with test scores, single-item measures scored at 100% accuracy. The NASA-demand score was high at 86%, followed by the overall NASA at 70% and the NASA-effort at 57%.

### Comparison of the Different Categories

By combining the data for each category of measure (see Table 6) sample sizes became sufficiently large to make meaningful statistical comparisons. Because the data did not fit normal distributions non-parametric tests were completed. For the three data sets summarized in columns 4–6, Kruskal–Wallis tests were completed for the five categories.

#### At Least 1-Significant Difference in Cognitive Load Detected

Comparing the five measures for this data there was an overall significant difference between the five categories (Kruskal–Wallis χ^2^ = 9.67, *df* = 4, *p* = 0.046). *Post hoc* tests using the Benjamini and Hochberg (1995) correction method indicated no significant pairwise differences. However, it is worth noting that subjective measures had an accuracy rate of 94% compared with Heart and Brain measures that were below 70%.

#### Matches With Task Performance

For this data there was a significant between-group difference (Kruskal–Wallis χ^2^ = 18.52, *df* = 4, *p* < 0.001). *Post hoc* tests indicated that both the subjective measures (*M* = 0.78, *SD* = 0.42, *p* = 0.002) and the eye measures (*M* = 0.78, *SD* = 0.42, *p* = 0.004) had significantly more matches with task performance than the brain measures (*M* = 0.32, *SD* = 0.47).

#### Matching Deviations

For this data there was a significant between-group difference (Kruskal–Wallis χ^2^ = 19.44, *df* = 4, *p* < 0.001). *Post hoc* tests indicated that both the subjective measures (*M* = 0.19, *SD* = 0.82, *p* < 0.001) and eye measures (*M* = 0.30, *SD* = 0.76, *p* = 0.006) had significantly less deviations from the task performances than the brain measures (*M* = 1.15, *SD* = 1.05).

In summary, the analyses provided in this section suggest that overall the subjective and eye measures were the most sensitive indicators of cognitive load differences across tasks. Clearly the least effective were brain measures. Measures associated with the skin and heart were located between the other indicators. Nevertheless, all subcategories of measures were able to detect some differences in cognitive loads, and also some matches with test scores. Only the heart measures on word problems failed to detect any significant differences or matches.

#### Task Comparisons

The data in Table 6 were examined in terms of task differences. The memory and arithmetic tasks recorded the best results. For both tasks, the combined measures were able to detect at least one cognitive load difference at the 90% level. For matches with performance, memory tasks achieved a 90% match, whereas arithmetic tasks had lower match rates at the 65% level. Simulations, object/shape manipulations and word tasks were overall at a lower level of accuracy, although overall matches were at least 50%.

#### Individual Measure Comparisons

The previous analysis in this section was on aggregated data. A closer look at individual measures was achieved by examining those specific measures (no other/combined categories were included) that had been used at least six times over the sample. By averaging the % scores for the data in columns 4 (at least one significant cognitive load difference) and 5 (matches with test performance) of Tables 1–5 the following ranking was found: single-item subjective measures (100%), NASA-demand (93%), Blink-rates (91.5%), NASA-overall (80%), HR (78.5%), GSR (73.5%), NASA-effort (71.5%), Pupil-diameter (68.5%), Alpha waves (67%), and HRV (50%). Each of these measures, across all five general categories scored at least at the 50% accuracy level. There were four scores above 90% indicating very high levels of accuracy at detecting cognitive load differences, but three of those four scores were subjective rather than physiological measures.

### Battery of Tests

As reported above nearly all studies (*N* = 30) included a battery of tests from within and/or between the categories (including subjective measures) to measure cognitive load. Examination of this data revealed that in all but three cases, at least one of the tests had an exact match with the expected number of cognitive load differences.

## Discussion

There is considerable evidence that supports the argument that changes in cognitive load can be detected by physiological measures that utilizes data signals collected from the heart and lungs, eyes, skin, and brain. Between and within these four categories, signals react differently according to the measure. For example, as cognitive load increases, pupil dilation is expected to increase, but HRV to decrease. From the perspective of a validity study, results should be consistent with the predictions made for each individual measure. The more studies find evidence for this prediction, the higher the construct validity of the measure. The data analyzed here indicate that physiological measures taken from all four parts of the body are capable of detecting changes in cognitive load generated by differences in task complexity. Of all the measures cataloged in Tables 1–4 only BP and skin temperature failed to find a significant difference. However, these measures were only reported in three studies and therefore these non-effects cannot be generalized. Overall measures associated with the heart and lungs, eyes, skin, and brain measures were all capable of finding significant cognitive load differences across tasks, providing a level of construct validity (see Borsboom et al., 2004). Therefore, in answer to RQ1 (*Do the physiological measures have construct validity in detecting changes in intrinsic cognitive load across tasks?)*, we conclude that nearly all the measures identified in this sample have some level of construct validity because of their capacity to detect changes frequently.

The information reported in Tables 1–4, 6 indicated that some physiological measures were more sensitive than others to finding cognitive load differences across tasks. The most sensitive physiological measures were blink rates, HR, pupil dilations, and alpha waves. Therefore, in answer to RQ2 (*How sensitive are the physiological measures in detecting changes in intrinsic cognitive load?)*, we conclude that the different measures were sensitive enough to identify differences in cognitive load but the levels of sensitivity varied significantly between measures.

By examining the different types of tasks used in these studies we found that memory and mental arithmetic tasks led to higher degrees of sensitivity for the cognitive load measures than simulations and object/shape manipulations. The former are short tasks relying completely on working memory capacity; whereas the latter are more authentic and specialized tasks that may depend more on long-term memory (e.g., knowledge of medical functions and procedures). Although no studies were included that manipulated stress and anxiety, these affective influences may have been generated by more authentic high-stakes tasks (e.g., surgery simulations) leading to confounding factors. Nevertheless, our evidence found that eye measures were more sensitive than other physiological tests for the more specialized tasks, perhaps due to the importance of information received visually. Clearly the type of task does impact on the different levels of sensitivity across the physiological measures and therefore overall validity (RQ3 – *Does the type of task impact on overall validity?*).

Although not the main aim of our study the sample provided data on subjective measures of cognitive load, which also showed validity. We found that compared with most of the physiological measures included in our sample, the subjective measures had a higher level of sensitivity. As reported above, single-item measures of cognitive load such as asking to rate the amount of effort, difficulty or demand experienced, were found to have the highest sensitivity. Only blink-rate data was at the same level. The favorable finding for subjective measures is interesting as in more recent time, CLT researchers have started to use more physiological tests for measuring cognitive load (Ayres, 2020). Many commentators have expressed concern over the high use of subjective measures suggesting more objective methods are required (see Schnotz and Kürschner, 2007; Kirschner et al., 2011).

Our data suggests that it may be premature to abandon subjective methods, but the question arises should we be more circumspect in using physiological measures? They are objective, but in our sample evidence emerged that many are not as sensitive as subjective measures. In only one study in the sample did a physiological measure (alpha signals) identify more variations in cognitive load than the subjective measure (overall NASA-TLX). It is also notable that nearly 30 years ago Paas and van Merriënboer (1994; see also Paas et al., 1994) found a self-rating measure of mental effort to be superior to HR data, which led to wide-scale adoption of subjective measures of effort and difficulty (see van Gog and Paas, 2008; Ayres, 2018). However, there are cases in the literature where subjective measures have also been found to lack sensitivity. For example, Lee et al. (2020) found pupil dilation can be more sensitive than self-rating measures. This was the case when the task included the management of emotions, and the confounding factors were well-controlled. Hence, the picture is not definitive and more examination of the influences on physiological measures is required, as reported next.

Clearly some of the physiological measures in this sample lacked sensitivity especially with specific types of tasks. However, this can be because these physiological measures did not optimally match with the type of task. The choice of the measure types is important for the question of sensitivity: some measures are more sensitive than others for specific tasks, even within the same category of physiological measures. For instance, in driving tasks, horizontal gaze dispersion showed a larger effect size than other eye-tracking measures (Wang et al., 2014). Another explanation described in the introduction is that there are a number of other conditions that can have a negative impact on physiological measures. For example, HR and eye measures can be influenced by participant motion (e.g., driving a car, see Lohani et al., 2019) EDA may be more sensitive to sudden rather than gradual changes, and some areas of the brain (e.g., the fronto-parietal attention network) are more conducive to measuring cognitive load than other areas. It is possible that some studies may have been influenced by such factors, and therefore some caution should be shown in interpreting the results unconditionally. In a review into using physiological measures of more wide-ranging causes of mental workload, Charles and Nixon (2019) concluded that there was no single preferred measure that could be used across all tasks and domains, but more evidence was emerging in how best to utilize each type. Our study to some extent supports this finding; however, we suggest further that until more research is completed, the best outcomes may be found in a well-chosen combination of tests.

In conclusion, we believe there is a solid case for including both physiological and subjective methods to measure cognitive load. Like other studies (see Johannessen et al., 2020) using a battery of different tests was found to be effective as in most cases at least one of the tests identified all the changes in cognitive load. Consequently, we suggest two points to take into account in using physiological measure to measure cognitive load: (1) select a measure based on the understanding of the given task; and (2) triangulate by combining different physiological measures as well as subjective measures. As our study showed, physiological measures can be valid in some setups (shown by results for RQ1), but also the sensitivity can vary across different measures depending on the task types (RQ2 and RQ3). Thus, careful selection of the right measures for the given task is essential. A thorough task analysis (e.g., cognitive task analysis) could be helpful in achieving this aim.

Triangulation is an effective research method to gain a comprehensive perspective and validation of data (Patton, 1999). Studies have shown that combining multiple physiological measures may present a higher sensitivity in measuring cognitive load than using a single measure (Van Orden et al., 2000; Aghajani et al., 2017). Combining physiological measures with subjective measures may show either positive convergence (Aldekhyl et al., 2018) or diverging sensitivity as aforementioned. However, such inconsistency does not necessarily represent an incredibility of data, but rather provides a deeper insight into the results (Patton, 1999).

We also found in this study that the nomenclature used by some authors was not always consistent with other authors. In a small number of studies, insufficient detail was provided in the method to make a definitive judgment on what exactly was used for the basis of the data calculations. Most notably this inconsistency was sometimes found in the GSR measures. For example, an ‘average’ GSR value would be reported without a clear indication of what part of the signal the average referred to. Although this did not impact on our overall findings, future studies should ensure that all necessary information is reported, and conform to a standardization of labels such as previously suggested by Fowles et al. (1981).

The sole focus of this study was on tasks that generated intrinsic cognitive load only. No attempt was made to include learning studies that would generate other types of cognitive load. Detecting changes in intrinsic load is important to CLT but so is extraneous cognitive load as CLT is predominantly interested in the impact of all types of cognitive load on learning. Hence, further research is required of this nature to explore validity during learning experiments that also featured between-subject experiments. As well as task complexity changes in cognitive load caused by other factors such as distractors (e.g., noise), and affective factors (e.g., emotion) should also be researched.

In addition, the studies in this sample were predominantly completed by young adults whose mean ages ranged from 20 to 30. However, two studies (see Tan et al., 2016; Abd Rahman et al., 2020) focused on elderly adults. Although these studies did not report any data that was inconsistent with the other studies in the sample, it is known that aging adults experience cognitive decline and are more susceptible to cognitive load variations (see van Gerven et al., 2000; Klencklen et al., 2017). Similarly, the sample did not include any young children. Subsequently, we cannot generalize our results to other age groups and therefore more research is needed in both older and younger populations to explore potential differences.

Our main focus was to examine the validity of using physiological measures of cognitive load. We conducted the study from a measurement and theoretical perspective especially that of cognitive load theory. It is worth mentioning, however, that there are real-world applications of measuring total cognitive load; often referred to as mental workload. For example, how to manage cognitive load during medical training and real-life practice (see Fraser et al., 2015; Johannessen et al., 2020; Szulewski et al., 2020) is critical. Similarly, in driving a car it can be advantageous from a safety perspective to be able to monitor the driver’s cognitive states and implement interventions accordingly (see Lohani et al., 2019; Meteier et al., 2021). When driving, real-time continuous data is required which can be collected through physiological measures, but using subjective measures is impossible. From this important perspective, physiological measures have a clear edge.

By analyzing a snapshot of studies taken from a recent 5-year period we have clearly not included some of the key studies from the period before this time. Although we cite a number of significant studies in our introduction, it was never our intention to go beyond our specified period. We wanted to investigate exactly what a contemporary collection of studies would reveal about measuring cognitive load. Our sample, like previous studies confirmed that different types of physiological measures are capable of measuring cognitive load.

There were a number of novel aspects to the study. Firstly, we included only studies that investigated the capacity of physiological measures to identify changes in intrinsic cognitive load by manipulating task complexity. Most studies in measuring cognitive load using physiological methods have not made this distinction. Secondly, by taking a broad sample we were able to compare a wide variety of physiological measures from four main categories, and to compare them with each other from both a construct validity and sensitivity perspective. An added bonus was that we were also able to benchmark them against a number of subjective rating scales. Thirdly, by examining the influence of task types, and the highlighting of technological precautions and other influences outlined in the literature review, we were able to identify some major factors that impact cognitive load measures.

In conclusion, we found that nearly all the physiological measures identified in this sample had some level of validity. However, there were wide variations in sensitivity to detect changes in intrinsic cognitive load, which was impacted by task specificity. In contrast, subjective measures generally had high levels of validity. We recommend that a battery of tests (physiological and/or subjective) are required to obtain the best indicators of changes in intrinsic cognitive load.

## Author Contributions

PA conducted the literature search and collected, compiled, and analyzed the data. PA, JL, FP, and JM designed the study and wrote the manuscript. All authors contributed to the article and approved the submitted version.

## Conflict of Interest

The authors declare that the research was conducted in the absence of any commercial or financial relationships that could be construed as a potential conflict of interest.

## Publisher’s Note

All claims expressed in this article are solely those of the authors and do not necessarily represent those of their affiliated organizations, or those of the publisher, the editors and the reviewers. Any product that may be evaluated in this article, or claim that may be made by its manufacturer, is not guaranteed or endorsed by the publisher.
